# A Family-Based Collaborative Care Model for Treatment of Depressive and Anxiety Symptoms in Perinatal Women: Results From a Pilot Study

**DOI:** 10.2196/45616

**Published:** 2023-04-13

**Authors:** Fallon Cluxton-Keller, Ardis Olson

**Affiliations:** 1 Department of Psychiatry Geisel School of Medicine at Dartmouth Lebanon, NH United States; 2 Department of Pediatrics Geisel School of Medicine at Dartmouth Lebanon, NH United States; 3 Department of Community and Family Medicine Geisel School of Medicine at Dartmouth Lebanon, NH United States

**Keywords:** anxiety, depression, family treatment, infant care, maternal health, parenting, pediatric primary care, perinatal anxiety, perinatal care, perinatal depression, video therapy, women's health

## Abstract

**Background:**

Untreated perinatal depression and anxiety can have detrimental consequences on family function. Logistical barriers prevent many perinatal women from accessing treatment, and these barriers are compounded for women residing in rural areas. This paper describes a Family-Based Collaborative Care Model (FBCCM) that is designed to bypass barriers to increase access to care for depressed and anxious perinatal women in rural regions of the United States. The FBCCM includes the following two components: (1) a 10-session video-delivered family therapy treatment for perinatal depression and anxiety and (2) a video-delivered infant care provider training on addressing the parenting needs of depressed and anxious mothers.

**Objective:**

This paper describes the feasibility of implementing the FBCCM with families and infant care providers. Findings are presented on the preliminary effectiveness of the video-delivered family therapy treatment in reducing maternal depressive and anxiety symptoms, and family conflict.

**Methods:**

This pilot study was carried out using an implementation-effectiveness hybrid trial design without a comparison group. Changes in maternal depressive symptoms, maternal anxiety symptoms, and family conflict were measured at posttreatment, 3 months, and 6 months later.

**Results:**

On average, mothers (n=24) attended 9.79 (SD 1.02) sessions. On average, their family members (n=24) attended 9.42 (SD 1.28) sessions. A total of 31 infant care providers attended the training on addressing the parenting needs of depressed and anxious mothers. Mothers reported a significant reduction in depressive symptoms (*P*<.001) and anxiety symptoms (*P*<.001) from baseline to the 6-month follow-up. Mothers reported a significant reduction in conflict (*P*<.001), and their family members also reported a significant reduction in conflict (*P*=.007) from baseline to the 6-month follow-up.

**Conclusions:**

The findings from this study provide support for the feasibility and preliminary effectiveness of the FBCCM. The findings will be used to inform a larger study of the FBCCM.

## Introduction

### Background

Perinatal depression and anxiety are increasing in the United States with prevalence rates ranging from 10% to 23% for depression [1-3] and 11% to 20% for anxiety [4,5]. Although the United States Preventative Task Force recommends for providers to screen women for perinatal depression and refer them for treatment [1], barriers (eg, no childcare or transportation) prevent women from getting treatment and under 25% initiate treatment [6,7]. These barriers are compounded for perinatal women in rural regions.

There is growing recognition that technology-based interventions for depression and anxiety bypass logistical barriers for pregnant and postpartum women [8-14]. Further, research has shown that technology-based interventions for perinatal depression and anxiety are feasible for use within routine perinatal care [8,9]. For example, MomMoodBooster2 has been shown to be an effective treatment option within routine perinatal care, especially when combined with universal depression screening and referral [8]. Although some technology-based treatments include some strategies to address instrumental support and relational health (ie, partner support website), the primary focus is cognitive-behavioral individual-level treatment for perinatal depression and anxiety [8,11-14].

Untreated perinatal depression and anxiety are precipitants and consequences of family conflict that worsens these symptoms [15-18]. Since mothers with perinatal depression tend to experience a mix of depressive and anxiety symptoms, the current COVID-19 pandemic has exacerbated their symptoms [19,20]. Perinatal depression and anxiety in combination with the current pandemic place demands on families that they are unprepared to meet, which can increase family conflict. Despite this, no evidence-based family therapy treatment exists for perinatal depression and anxiety [15,21,22].

This study was conducted during the COVID-19 pandemic and explored the feasibility and preliminary effectiveness of a Family-Based Collaborative Care Model (FBCCM) to address perinatal depression and anxiety. The FBCCM includes following two components: (1) a video-delivered family therapy treatment for depressed and anxious perinatal women receiving obstetrics care in rural clinics and (2) training for infant care providers on addressing the parenting needs of depressed and anxious mothers. The FBCCM uses technology to increase access to treatment for perinatal depression and anxiety and training for infant care providers in rural areas.

### Objectives

This paper has 2 objectives. The first objective is to present findings on the feasibility of the FBCCM for use with families and infant care providers. The second objective is to present findings on the preliminary effectiveness of the video-delivered family therapy treatment in reducing maternal depressive symptoms and anxiety symptoms, and family conflict. We hypothesized that (1) perinatal women will report significant reductions in depressive symptoms and anxiety symptoms from baseline to the 6-month follow-up, and (2) families will report a significant reduction in conflict from baseline to the 6-month follow-up.

## Methods

### Study Design

This implementation effectiveness hybrid trial, without a comparison group, tested both clinical interventions and implementation strategies [23]. The first objective pertains to implementation aspects of the design and reflects strategies to explore the feasibility of implementing the FBCCM with families receiving routine care in obstetrics clinics and infant care providers. The second objective pertains to clinical effectiveness aspects of the design to explore preliminary impacts on maternal and family outcomes.

### Ethics Approval

This study has been approved by an institutional review board (IRB) at an academic medical center in New England (02000646).

### Framework of FBCCM

Our research on perinatal depression guided the development of the FBCCM that includes 2 components. The first component is a video-delivered family therapy treatment (Resilience Enhancement Skills Training [REST]) [24-26] that is summarized in this section. REST is informed by Dialectical Behavior Therapy (DBT) Skills Training [27] and includes systemic interventions [28] to address conflict. Standard DBT is based on cognitive-behavioral therapy and includes individual therapy, skills training, and coaching, and it is effective for use in populations with severe depressive symptoms [29-31]. DBT skills training targets cognitive, emotional, and behavior regulation [27]. REST includes a total of 10 weekly, 30-minute interactive sessions that are delivered by a clinician using Health Insurance Portability and Accountability Act–compliant video technology (VCT) to families. Families attend sessions by clicking links on their cell phones, tablets, or computers from home. To date, REST has been delivered using 2 types of Health Insurance Portability and Accountability Act VCT: Vidyo [32] and WebEx [33].

The results of the preliminary pilot study of REST with home-visited families showed that it significantly reduced maternal depressive symptoms and family conflict [24]. Although the participants in this study had an overall higher level of education and greater financial security than those who participated in other studies of REST [24-26], the severity levels for maternal depressive symptoms and family conflict are similar. This study expands the existing research on REST to explore its impact on perinatal anxiety.

The first author delivered REST to families in this study using Vidyo [32] and Cisco WebEx [33]. The selection of VCT was dependent on the participant’s bandwidth consumption limits. This study used the same technology training procedures for REST participants that were used in other studies [24-26].

The second component of the FBCCM is a 1-hour video-delivered training session provided by the second author to infant care providers on addressing the parenting needs of depressed and anxious mothers within the context of well-child visits. The training was offered during lunchtime to increase the likelihood of provider attendance. The training used elements of the second author’s guide on addressing maternal depression in primary care [34], cited by the American Academy of Pediatrics [35,36]. The established training was expanded to include guidance on addressing the parenting needs of anxious mothers. The training primarily focused on expanding maternal knowledge of developmentally appropriate expectations, validating parenting efforts, and responding to infant fussiness.

### Eligibility Criteria

The study population included mothers at least 18 years of age in any trimester of pregnancy and up to three months postpartum, and their adult family members receiving perinatal care in 2 participating obstetrics clinics in rural regions of New England. This study included a 2-phase eligibility screen process for mothers. Mothers routinely complete the *Patient Health Questionnaire-2* *item* [37] and *Generalized Anxiety Disorder-2 item* [38,39] in the patient portal on electronic tablets when they check in for perinatal care appointments. In phase 1, mothers with *Patient Health Questionnaire-2* *item* scores of ≥2 [40] or *Generalized Anxiety Disorder-2 item* scores of ≥3 [39] were automatically directed to an IRB-approved study information sheet (included an overview of the study goals, video-delivered family therapy treatment, and potential benefits and risks) to read that was followed by a question on whether or not they wanted to be contacted by the first author to learn more about the study. Mothers who selected “Yes” to the question entered their contact information in a textbox. The electronic medical record system automatically sent the first author a notification with the contact information of mothers who requested to be contacted about the study. Mothers who did not want to be contacted about the study received standard services (eg, referral to mental health provider) through the obstetrics clinic where they were receiving perinatal care.

The first author called mothers within 48 hours of receipt of their requests to provide more details on the study and ask if they wanted to proceed with the phase 2 eligibility screen. The first author secured electronic consent for the phase 2 eligibility screen from mothers and administered the measures. Each mother was asked to select a “family member” (defined as her adult relative or current intimate partner) with whom she had conflict who could potentially participate with her in the study. The Family Environment Scale-Conflict (FES-C) subscale [41] was administered to mothers to assess the level of conflict between the mother and the selected family member. Mothers with FES-C scores of at least four (indicative of moderate to high conflict) without domestic violence as measured by the Abuse Assessment Screen [42] were eligible for participation. The Beck Depression Inventory-Second Edition (BDI-II) [43] and State-Trait Anxiety Inventory-State Anxiety (STAI-S) scale [44] were administered to mothers to detect severity of symptoms. Mothers with BDI-II scores below 54 without suicidal ideation were eligible for study participation. Participants had to be fluent in English since the intervention materials were written in English. Participants had to have consistent internet access (ie, subscribe to an internet service provider without weekly disruptions in service) on a cell phone, tablet, or computer equipped with a camera and microphone to participate in sessions. Mothers on stable doses (eg, at least 3 months) of psychiatric medications were eligible for study participation. Mothers with current individual therapy or a history of DBT were not eligible for study participation. The first author referred ineligible mothers to appropriate services in the community.

Infant care providers, willing physicians, resident physicians, and nurse practitioners who provided infant care at participating pediatric and family medicine clinics were eligible for study participation. The second author used video communication technology (Zoom) to deliver presentations to infant care providers that included an overview of the study goals, study participation, and description of the FBCCM (infant care provider training and family therapy treatment) potential benefits and risks. Infant care providers were informed that mothers, some of whom were served by their clinics, would be recruited for study participation and this confidential information could only be shared with them by the mother. Infant care providers were informed that the training would be provided to help mothers who are participating in the study and any other mothers with depression and anxiety who are served in their clinics.

### Recruitment and Consent Procedures

The first author secured electronic consent of the IRB-approved consent form for the phase 2 eligibility screen from 89 willing mothers. Of these 89 screened mothers, 29 of them were ineligible (n=16 low family conflict; n=7 receiving therapy; n=5 inconsistent internet access; and n=1 upcoming move to another state). The first author referred ineligible mothers to appropriate services in the community. Of the 60 eligible mothers, 21 of them decided not to enroll in the study due to busy schedules.

The first author secured electronic consent of the IRB-approved consent form for study enrollment from 39 willing mothers and their 29 willing family members. It is important to note that 10 mothers enrolled in the study without family members, and we plan to prepare a separate paper with the data on this subgroup of the sample. Of the 29 families who enrolled in the study, 3 of them dropped out before the first session due to busy schedules.

Infant care providers from 5 participating clinics were invited to attend the training. The second author secured electronic consent of the IRB-approved consent form for study enrollment from 9 willing infant care providers to complete a survey prior to the training. The 9 infant care providers were from 3 of the clinics.

### Data Collection

Participants completed a web-based baseline questionnaire in research electronic data capture [45]. The maternal and family member questionnaire included demographic items (eg, age, race, gender, highest level of education, and employment). The infant care provider questionnaire included demographic items (eg, age and gender). It also included items that pertained to current clinic practices for screening for maternal depression and anxiety with response options of “yes” or “no.” It included items on perceived job responsibilities for addressing maternal depression and anxiety within the context of well-child visits with response options that ranged from 1 “strongly disagree” to 4 “strongly agree.”

### Feasibility

The first author calculated the number of REST sessions attended by each participant and the number of families who completed REST. The second author recorded the number of infant care providers who attended the training.

The first author closely monitored maternal depressive and anxiety symptoms (safety) and family conflict (tolerability) during the treatment phase. Mothers completed the BDI-II [43] after session numbers 2, 4, 6, and 8. The time points for the BDI-II align with standard guidelines for monitoring moderate to severe perinatal depression during treatment [46,47]. Since this was the first REST study that enrolled perinatal women with moderate to severe anxiety, the STAI-S [44,48,49] was also administered to mothers at these 4 time points in the treatment phase to closely monitor anxiety symptoms. The safety protocol outlined referral procedures for mothers with increased BDI-II or STAI-S scores from baseline, indicative of increased moderate to severe symptoms, for more intensive treatment services. The protocol specified that mothers with suicidal ideation were immediately connected to emergency services. The first author administered the FES-C subscale [41] to each mother and her family member separately after session numbers 4 and 8 to assess tolerability. The FES-C is designed to measure conflict in the past month [41]. Each respondent was directed to respond to the items based on experiences that occurred within the past month with the family member who participated in the study. The tolerability protocol outlined referral procedures to services in the community for families with increased conflict or sustained high conflict from baseline. This protocol also specified safety planning and referral procedures if domestic violence was reported by a mother or her family member.

### Preliminary Effectiveness of REST

Mothers and family members completed web-based measures in research electronic data capture at baseline, postintervention (within a week after the final session), and 3-month and 6-month follow-ups.

The STAI-S [44] was used to measure maternal anxiety in this study. It is a reliable and valid measure in perinatal women [48,49]. It includes 20 items that are rated by symptom intensity. STAI-S scores range from 20 to 80, and scores of at least 38 indicate moderate to high anxiety in perinatal women [50-52].

The BDI-II [43] was used to measure maternal depression in this study. It has established reliability and validity in perinatal women [53-55]. It includes 21 items to assess depressive symptom severity by intensity and frequency, including suicidal ideation. The BDI-II scores range from 0 to 63, and scores of at least 20 indicate moderate to severe depressive symptoms [43].

The NIH Toolbox *Perceived Hostility Survey Ages 18+* (PHS) [56] was used as the family conflict outcome measure in this study. It is a reliable and valid measure for conflict-based communication [56]. It includes 8 items, each have a 5-point scale with response options that range from 1 “never” to 5 “always” [56]. PHS scores range from 8 to 40, and scores of at least 16 indicate moderate to high conflict in perinatal women [57]. Each respondent was directed to respond to the items based on experiences in the past month with the family member who participated in the study.

### Statistical Methods

#### Participant Characteristics

Univariate statistics were used to characterize families at baseline and infant care providers.

#### Feasibility

Ratios for actual to expected number of sessions (10 sessions) were calculated for each family in REST. Means and SDs were calculated for sessions attended by mothers and family members. High retention was defined as families attending ≥80% of sessions. The number of infant care providers who attended the training session was calculated.

For safety of REST for mothers, means and SDs were calculated for the BDI-II and the STAI-S from baseline through each clinical monitoring time point (session numbers 2, 4, 6, and 8). Wilcoxon signed rank tests were used to assess changes in BDI-II and STAI-S scores from baseline through session number 8. For tolerability of REST for families, means and SDs were calculated for the FES-C from baseline through each clinical monitoring time point (session numbers 4 and 8). Maternal and family member scores were calculated separately for the FES-C at each clinical monitoring time point. Wilcoxon signed rank tests were used to assess changes in FES-C scores from baseline through session number 8 for mothers and family members separately.

#### Preliminary Effectiveness of REST

Given the small sample size of mothers, the Friedman test was used to assess changes in maternal depressive symptoms (BDI-II scores) and maternal anxiety symptoms (STAI-S scores) from baseline to the follow-up time points (postintervention, 3-month follow-up, and 6-month follow-up). For significant results, post hoc pairwise comparison analyses were conducted using the Wilcoxon signed rank test with a Bonferroni correction applied, resulting in a significance level set at *P*=.008. Evidence of effectiveness will be demonstrated at the 6-month follow-up by significant reductions in maternal depressive symptoms on the BDI-II and maternal anxiety symptoms on the STAI-S.

Family conflict was analyzed in mothers and their family members separately. Given the small sample size of mothers and small sample size of family members, the Friedman test was used to assess changes (PHS scores) from baseline to the follow-up time points (postintervention, 3-month follow-up, and 6-month follow-up). For significant results, post hoc pairwise comparison analyses were conducted using the Wilcoxon signed rank test with a Bonferroni correction applied, resulting in a significance level set at *P*=.008. Evidence of effectiveness will be demonstrated at the 6-month follow-up by significant reductions in family conflict on the PHS.

## Results

### Participant Characteristics

Of the 26 families who started REST, 24 families contributed follow-up data. The baseline characteristics for these 24 families are included in Table 1. Mothers’ ages ranged from 26 to 40 years old. Mothers either selected their partners or spouses to participate with them in the study; 88% (21/24) of mothers were married. Family members’ ages ranged from 27 to 49 years old. About 96% (n=23) of mothers were pregnant at baseline. About 63% (n=15) of mothers were in the second trimester of pregnancy, and the remainder of them were in the third trimester. Half were first-time mothers. About 46% (n=11) of mothers had moderate to severe anxiety symptoms and moderate to severe depressive symptoms. Half of mothers had moderate to severe anxiety symptoms and mild depressive symptoms. One mother had severe depressive symptoms and mild anxiety symptoms.

The characteristics for the 9 infant care providers are included in Table 2. All providers identified as pediatricians, and 44% (4/9) of them were resident physicians. Prior to the training, one-third of providers reported they had not received sufficient education about maternal depression and how to address it in practice. Over half of providers (56%, 5/9) reported they had not received sufficient education about maternal anxiety and how to address it in practice. About 78% (7/9) of providers agreed that family social determinants of health limited what they could do to help depressed mothers and anxious mothers.

**Table 1:** Family baseline characteristics.

		Mothers (N=24)	Family members (N=24)
Age (years), mean (SD)		32.79 (3.08)	34.13 (4.84)
**Race, n (%)**			
	Asian	2 (8)	0 (0)
	More than one race	1 (4)	1 (4)
	White	21 (88)	23 (96)
**Highest level of education, n (%)**			
	High school diploma or General Educational Diploma	1 (4)	2 (8)
	College degree	8 (33)	11 (46)
	Graduate school degree	15 (63)	11 (46)
Employed, n (%)		18 (75)	22 (92)
Family conflict score^a^, mean		55.8	54.4
Maternal depression score^b^, mean (SD)		20 (7.62)	N/A^c^
Maternal anxiety score^d^, mean (SD)		43.63 (7.37)	N/A

^a^Perceived Hostility Survey Ages 18+ (PHS) [56] uncorrected T-Score from the NIH Toolbox Raw Score to T-Score Conversion Table.

^b^Beck Depression Inventory-Second Edition (BDI-II) scores of 20-28 indicate moderate depressive symptoms [43].

^c^N/A: not applicable.

^d^State-Trait Anxiety Inventory-State Anxiety (STAI-S) [44] scale scores of at least 38 indicate clinically significant anxiety [50].

**Table 2:** Infant care provider characteristics.

				Infant care providers (N=9)
Age (years), mean (SD)				35.56 (7.49)
Female, n (%)				7 (78)
**Race, n (%)**				
	More than one race			1 (11)
	White			8 (89)
Clinic implements postpartum depression screens, n (%)				6 (67)
**Job responsibilities for maternal depression, n (%)**				
	**Recognition of postpartum depression**			
		Strongly agree	6 (67)	
		Agree	3 (33)	
	**Addressing parenting issues that pertain to maternal depression**			
		Strongly agree	6 (67)	
		Agree	3 (33)	
Clinic implements postpartum anxiety screens, n (%)				2 (22)
**Job responsibilities for maternal anxiety, n (%)**				
	**Recognition of postpartum anxiety**			
		Strongly agree	4 (44)	
		Agree	5 (56)	
	**Addressing parenting issues that pertain to maternal anxiety**			
		Strongly agree	5 (56)	
		Agree	4 (44)	

### Feasibility

Of the 26 families who started REST, 2 families dropped out prior to the third session due to busy schedules. One family attended 5 sessions, reported they resolved their conflict, and they did not need more sessions. Nearly all mothers (96%; 23/24) attended all 10 sessions. On average, mothers attended 9.79 (SD 1.02) sessions. A few family members had to miss sessions due to work commitments. On average, family members attended 9.42 (SD 1.28) sessions. Overall, 83% of participants attended all 10 sessions.

The training on addressing maternal depression and anxiety in well-child visits was offered to infant care providers at 5 participating clinics. A total of 31 infant care providers attended the training.

**Figure 1:**
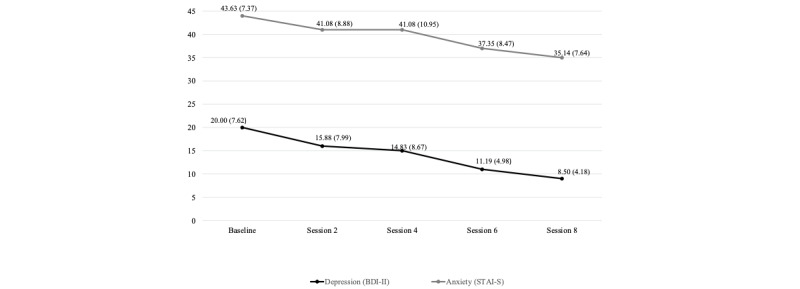
Changes in maternal depressive and anxiety symptoms from baseline through the treatment phase. BDI-II: Beck Depression Inventory-Second Edition; STAI-S: State-Trait Anxiety Inventory-State Anxiety scale.

The results indicate that REST is safe for mothers. Mothers reported a significant reduction in depressive symptoms (*Z*=–3.95, *P*<.001) and a significant reduction in anxiety symptoms (*Z*=–3.49, *P*<.001) from baseline through session 8. No mothers required referrals to intensive mental health services. Figure 1 shows the changes in maternal depressive symptoms and maternal anxiety symptoms from baseline through the treatment phase.

**Figure 2:**
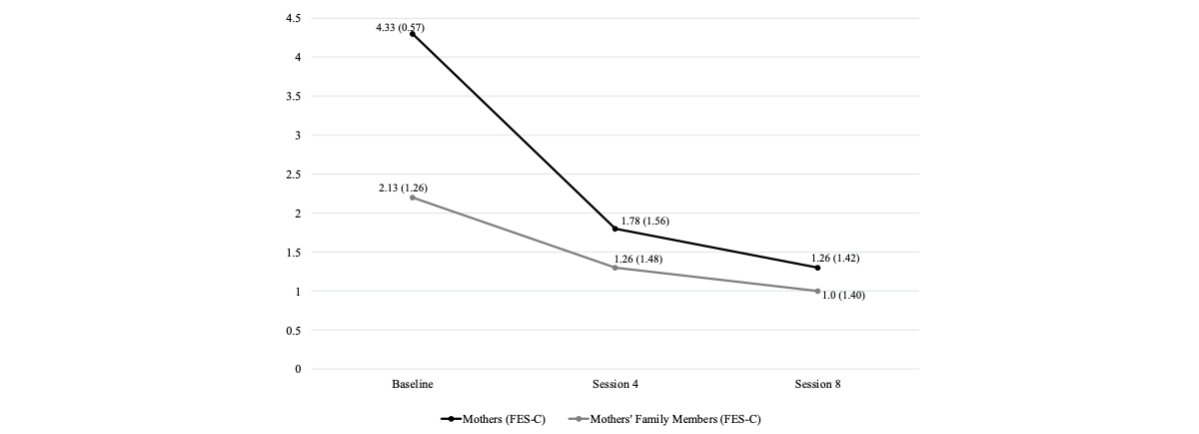
Tolerability of Resilience Enhancement Skills Training. FES-C: Family Environment Scale-Conflict subscale.

The results indicate that REST is well tolerated by families. No families were removed from the study due to increased family conflict on the FES-C or domestic violence. Mothers reported a significant reduction in family conflict on the FES-C (*Z*=–4.16, *P*<.001) and their family members also reported a significant reduction in family conflict on the FES-C (*Z*=–2.42, *P*=.02) from baseline through session number 8. Figure 2 shows the changes in family conflict in mothers’ FES-C scores and family members’ FES-C scores from baseline through the treatment phase.

### Preliminary Effectiveness of REST

Of the 48 participants who completed the baseline measure, 47 of them also completed the postintervention measure. One mother’s family member was unable to complete the postintervention measure but completed the 3-month and 6-month follow-up measures. Two couples (n=4 individuals) were lost to follow-up after completing the postintervention measure.

Table 3 includes the changes in maternal depressive and anxiety symptoms from baseline to each of the 3 follow-up time points. Mothers reported a significant decrease in depressive symptoms over time (*χ*^2^_3,22_=27.26, *P*<.001). There were statistically significant reductions in maternal depressive symptoms from baseline to postintervention (*Z*=–4.06, *P*<.001), baseline to the 3-month follow-up (*Z*=–3.87, *P*<.001), and baseline to the 6-month follow-up (*Z*=–3.82, *P*<.001). Although the significant reduction in maternal depressive symptoms was sustained at the 6-month follow-up, there were no significant differences in maternal BDI-II scores from postintervention to the 3-month follow-up (*Z*=–1.15, *P*=.25), postintervention to the 6-month follow-up (*Z*=–1.22, *P*=.22), and the 3-month follow-up to the 6-month follow-up (*Z*=–0.61, *P*=.54).

**Table 3:** Changes in maternal depressive and anxiety symptoms by time point.

	Baseline (N=24)	Postintervention (N=24)	*P* value	3-month follow-up (N=22)	*P* value	6-month follow-up (N=22)	*P* value
BDI-II^a^, mean (SD)	20 (7.62)	6.96 (2.77)	<.001	8.46 (4.19)	<.001	8.27 (5.11)	<.001
STAI-S^b^, mean (SD)	43.63 (7.37)	33.17 (6.94)	<.001	33.73 (7.97)	<.001	33.77 (7.56)	<.001

^a^BDI-II: Beck Depression Inventory-Second Edition scores.

^b^STAI-S: State Trait Anxiety Inventory- State Anxiety scale.

Mothers reported a significant decrease in anxiety symptoms over time (*χ*^2^_3,22_=25.29, *P*<.001). There were statistically significant reductions in maternal anxiety symptoms from baseline to postintervention (*Z*=–4.03, *P*<.001), baseline to the 3-month follow-up (*Z*=–3.67, *P*<.001), and baseline to the 6-month follow-up (*Z*=–3.81, *P*<.001). Although the significant reduction in maternal anxiety symptoms was sustained at the 6-month follow-up, there were no significant differences in maternal STAI-S scores from postintervention to the 3-month follow-up (*Z*=–0.42, *P*=.68), postintervention to the 6-month follow-up (*Z*=–0.15, *P*=.88), and the 3-month follow-up to the 6-month follow-up (*Z*=–0.14, *P*=.89).

Table 4 includes changes in family conflict, measured separately using mothers’ PHS scores and their family members’ PHS scores, from baseline to each of the 3 follow-up time points. Mothers reported a significant decrease in conflict with their participating family members over time (*χ*^2^_3,22_=26.66, *P*<.001). There were statistically significant reductions in mothers’ PHS scores from baseline to postintervention (*Z*=–3.90, *P*<.001), baseline to the 3-month follow-up (*Z*=–2.98, *P*=.003), and baseline to the 6-month follow-up (*Z*=–3.41, *P*<.001). Although the significant reduction in maternal reported family conflict was sustained at the 6-month follow-up, there were no significant differences in maternal PHS scores from postintervention to the 3-month follow-up (*Z*=1.31, *P*=.19), postintervention to the 6-month follow-up (*Z*=–0.79, *P*=.43), and the 3-month follow-up to the 6-month follow-up (*Z*=–0.57, *P*=.57).

Family members also reported a significant decrease in conflict with mothers over time (*χ*^2^_3,21_=17.65, *P*<.001). There were statistically significant reductions in family member reported conflict from baseline to postintervention (*Z*=–2.81, *P*=.005), baseline to the 3-month follow-up (*Z*=–3.32, *P*<.001), and baseline to the 6-month follow-up (*Z*=–2.67, *P*=.007). Although the significant reduction in family member reported conflict was sustained at the 6-month follow-up, there were no significant differences in family member PHS scores from postintervention to the 3-month follow-up (*Z*=–1.42, *P*=.16), postintervention to the 6-month follow-up (*Z*=–0.16, *P*=.88), and the 3-month follow-up to the 6-month follow-up (*Z*=1.44, *P*=.15).

**Table 4:** Changes in family conflict by participant type and time point.

	Baseline (N=48)	Postintervention (N=47)	*P* value	3-month follow-up (N=44)	*P* value	6-month follow-up (N=44)	*P* value
Maternal PHS^a^, mean	55.8	48.1	<.001	49.8	.003	48.1	<.001
Family member PHS^a^, mean	54.4	51.4	.005	48.1	<.001	49.8	.007

^a^Perceived Hostility Survey Ages 18+ uncorrected mean T-Score from the NIH Toolbox Raw Score to T-Score Conversion Table.

## Discussion

### Principal Results

The findings from this study support the feasibility and preliminary effectiveness of REST in reducing maternal depressive symptoms, maternal anxiety symptoms, and family conflict from baseline through the 6-month follow-up. Mothers reported a significant reduction in depressive symptoms (*P*<.001) and a significant reduction in anxiety symptoms (*P*<.001) from baseline to the 6-month follow-up. This study is the first to explore REST’s potential effectiveness in reducing maternal anxiety. We plan to conduct more research to determine REST’s effectiveness in reducing maternal anxiety.

Mothers reported a significant reduction in conflict with their family members (*P*<.001) from baseline to the 6-month follow-up. Family members reported a significant reduction in conflict with mothers from baseline to the 6-month follow-up (*P*=.007). More studies are needed to show that REST is an effective treatment, especially in ethnically diverse families. A preliminary pilot study of REST showed that it was feasible and acceptable for socioeconomically disadvantaged families [24]. A pilot randomized trial is currently underway that is testing REST’s impacts on depression and family function in a more diverse population of families [26], and the results will be published in a subsequent journal paper.

A total of 31 infant care providers attended the training on addressing the parenting needs of depressed and anxious mothers. Given that the providers are overburdened with clinical responsibilities that have multiplied due to the current pandemic, it is not surprising that only 9 of them agreed to complete the questionnaire prior to the training. Of these 9 infant care providers, 67% (n=6) of them reported that mothers are screened for depression at their clinics, and 22% (n=2) of them reported that mothers are screened for anxiety at their clinics. Although the infant care providers acknowledged that it is important to screen mothers for anxiety; the lack of available treatment resources in rural regions has likely inhibited clinic managers’ decisions to require routine screening procedures for maternal depression and anxiety. Regardless of clinic decisions to implement routine screening procedures, mothers may still disclose depressive symptoms and anxiety symptoms during well-child visits and infant care providers need to know how to best help them. This study offered these providers convenient and easily accessible training on addressing the parenting needs of depressed and anxious mothers.

### Comparison With Prior Work

The current findings for REST can be compared to those of previous research [24]. The feasibility results that pertain to family retention, session attendance, safety, and tolerability of REST are consistent with those of previous research on REST [24]. REST was primarily developed for use with families enrolled in early childhood home visiting programs. Although the sample of families in this study was not enrolled in home visiting, the findings on REST’s impacts on maternal depression and family conflict are consistent with the findings for the home visited families [24]. The current and previous results [24] showed that mothers experienced significant reductions in depressive symptoms, and families experienced significant reductions in conflict. The current findings strengthen the previous findings in that the current findings show that REST’s impact was sustained for 6 months after the final session.

The characteristics of the infant care providers who completed the questionnaire prior to the training are similar to those who participated in the second author’s previous research [58]. The study took place during the COVID-19 pandemic and many of the participating clinics were rapidly transitioning to electronic administration of patient clinical screening measures and video-delivered services, which required providers to quickly adjust to these new practices. Although infant care providers were very busy adapting to these new changes, they prioritized the training in an effort to increase their knowledge on addressing the parenting needs of depressed and anxious mothers. The training time and duration, 1 hour at lunchtime, and video-based format likely made it convenient and easy for them to attend it.

### Limitations

This study is not without limitations. First, the sample sizes were small for families and infant care providers. For this reason, the findings should be interpreted with caution. Second, few ethnically diverse families and infant care providers participated in the study. Thus, the results may not be generalizable to more diverse populations. Although rural areas of the United States are less ethnically diverse than urban areas [59], we plan to conduct future research on the effectiveness of FBCCM with ethnically diverse populations. Third, this pilot study did not include a comparison group. It was not feasible to include a comparison group in this study. We plan to conduct a larger study that will include comparison groups in order to assess differences in outcomes for FBCCM participants.

### Conclusions

This paper included findings that support the feasibility of the FBCCM and preliminary effectiveness of the family therapy treatment component of this model. These findings are important in justifying a larger study. Our future research will focus on testing the impact of the FBCCM in improving outcomes for families and infant care providers.

## Abbreviations

BDI-II: Beck Depression Inventory-Second Edition
DBT: Dialectical Behavior Therapy
FBCCM: Family-Based Collaborative Care Model
FES-C: Family Environment Scale-Conflict
IRB: institutional review board
PHS: Perceived Hostility Survey Ages 18+
REST: Resilience Enhancement Skills Training
STAI-S: State-Trait Anxiety Inventory-State Anxiety scale
VCT: compliant video technology
